# The influence of altered lower-limb muscle strength on dynamic plantar pressure distribution in participants who underwent anterior cruciate ligament reconstruction

**DOI:** 10.3389/fspor.2025.1569129

**Published:** 2025-08-20

**Authors:** Karolína Matov, Michal Bozděch, Marta Gimunová, Martin Komzák, Marek Dostál, Miloslav Maršálek, Tomáš Vespalec, Milan Mojžíš, Tomáš Vodička

**Affiliations:** ^1^ Department of Physical Activities and Health Sciences, Masaryk University, Faculty of Sports Studies, Brno, Czechia; ^2^ Department of Physical Education and Social Sciences, Masaryk University, Faculty of Sports Studies, Brno, Czechia; ^3^ Department of Biophysics, Masaryk University, Faculty of Medicine, Brno, Czechia; ^4^ Orthopedic Clinic, University Hospital Brno, Brno, Czechia

**Keywords:** anterior cruciate ligament reconstruction, plantar pressure, muscle strength, isokinetic dynamometry, return-to-the-sport criteria, asymmetries, injury

## Abstract

**Introduction:**

Deficits in lower-limb muscle strength and altered gait mechanics are common after anterior cruciate ligament reconstruction (ACL). While isokinetic strength testing is widely accepted in return-to-sport assessment, the role of plantar pressure analysis in detecting compensatory gait strategies remains underexplored.

**Methods:**

This study included 10 male patients (30.27 ± 5.59 years; 178.37 ± 6.30 cm; 84.85 ± 10.74 kg) who underwent ACL reconstruction using bone–patellar tendon–bone autografts. Assessments were performed preoperatively and at 3 and 6 months postoperatively, evaluating isokinetic knee strength and plantar pressure distribution during barefoot level walking. Non-parametric Friedman tests with Kendall's W assessed temporal differences, followed by Conover *post hoc* tests with Bonferroni correction. Spearman's rank correlation examined associations between muscle strength and plantar pressure.

**Results:**

Significant deficits in extensor strength were found at both postoperative time points compared to preoperative levels (both *p* < .001), with improvement at six months vs. three (*p* < .001). Flexor strength showed a similar but less pronounced recovery (*p* = .005). Plantar pressure analysis revealed changes relative to baseline: reduced hindfoot contact area at six months (*p* = .035), decreased midfoot maximum force at three (*p* = .047) and six months (*p* = .026), and lower peak pressure under the fifth metatarsal head at six months (*p* = .035). No significant correlations emerged between muscle strength and plantar pressure parameters.

**Discussion:**

These findings suggest plantar pressure analysis may complement return-to-sport evaluation by revealing hindfoot asymmetries persisting despite strength recovery. However, as dynamic plantar pressure parameters do not reflect quadriceps or hamstring status reliably, they cannot replace standard tests like isokinetic dynamometry. Combining plantar pressure metrics with conventional strength and functional assessments may better identify residual gait deficits and guide targeted rehabilitation to lower reinjury risk.

## 1 Introduction

The anterior cruciate ligament (ACL) is the most frequently injured ligament of the knee, particularly in athletes participating in pivoting and cutting sports (1), with an incidence rate ranging from 30 to 80 cases per 100,000 individuals (2, 3). The incidence of ACL injuries has sharply increased in recent years, rising by approximately 58% when comparing data from 2009 to 2016 to that from 2000 to 2005 (4). These injuries impose significant consequences, including medical expenses, decreased quality of life, and increased risk of early-onset knee osteoarthritis (5, 6).

ACL reconstruction remains the primary treatment for restoring knee stability, facilitating return-to-sport (RTS) (7), and minimizing osteoarthritis risk (1). Following ACL reconstruction, patients typically undergo rehabilitation for 6 to 12 months, a critical period for achieving RTS (2). Ardern et al. (8) reported that only 62% to 81% of individuals return to sports at their pre-injury level, with a significantly smaller proportion (24%−44%) resuming competitive-level activities (9). A major concern for athletes returning to sports after ACL reconstruction is the elevated risk of a second ACL injury, which may involve either graft failure or injury to the contralateral ACL (10, 11). However, despite advances in surgical techniques and rehabilitation, the reinjury rate remains alarmingly high, ranging from 5.8% to 27% (2, 12).

Substantial research has been dedicated to establishing RTS criteria aimed at minimizing the risk of reinjury. These criteria are multifactorial and encompass a combination of functional performance tests, self-reported outcomes on rehabilitation progress (7), and the time elapsed since surgery—commonly set at a minimum of six months (13), with a more widely accepted standard set at nine months (14). Additionally, psychological readiness, including fear of reinjury (15) and confidence levels (16), as well as muscle strength assessments, are critical components of RTS evaluation.

Muscle strength testing plays a particularly pivotal role in RTS criteria and is commonly assessed using hop tests and isokinetic dynamometry of the knee joint. Among the various RTS measures, isokinetic dynamometry of the quadriceps and hamstring muscles is widely recognized as the gold standard for strength evaluation (17). Achieving adequate muscle strength—particularly in the operated limb—is considered essential for ensuring joint stability, minimizing gait asymmetries, and reducing the risk of reinjury (18).

Recent studies (19–21) indicate that meeting RTS criteria after ACL reconstruction may not reliably prevent a second injury. This finding aligns with prior research (22, 23) suggesting that traditional RTS criteria may be insufficient for evaluating dynamic ACL function. Moreover, Knezevic et al. (24) highlighted the limitations of conventional strength testing, which often relies on single-plane assessments and may not fully reflect the multidirectional demands of sport-specific movements.

In this context, plantar pressure assessment during gait has been proposed as a complementary tool for evaluating functional recovery after ACL reconstruction. Prior studies (23) have shown that altered plantar pressure patterns may persist despite meeting traditional RTS benchmarks, offering additional insight into residual gait asymmetries. These gait characteristics could help refine RTS decision-making and potentially reduce the risk of reinjury.

However, limited knowledge exists regarding the relationship between muscle strength deficits post-ACL reconstruction and plantar pressure distribution, which may contribute to altered gait patterns. A better understanding of these associations is important, as dynamic plantar pressure assessment could serve as an additional RTS criterion and help identify individuals at risk of secondary ACL injuries.

The purpose of this study was to explore whether insufficient muscle strength following ACL injury is associated with changes in dynamic plantar pressure distribution and gait patterns in individuals post-ACL reconstruction. We hypothesized that limited quadriceps and hamstring strength following ACL reconstruction would be associated with persistent abnormalities in plantar pressure distribution—particularly in the hindfoot and midfoot regions—reflecting compensatory gait strategies. Furthermore, we expected that lower-limb muscle strength would correlate with plantar pressure parameters, indicating a functional link between isokinetic strength and gait-related loading patterns.

## 2 Materials and methods

### 2.1 Study participants

For the purpose of this study we selected 10 male participants (age 30.27 ± 5.59 yo; height 178.37 ± 6.30 cm; body mass 84.85 ± 10.74 kg) indicated to ACL reconstruction. Participants were selected by orthopedics physicians at the orthopedics department at The University Hospital Brno, Brno, Czech Republic and Hospital of Znojmo, Znojmo, Czech Republic.

Participants were non-professional and recreational athletes, indicated for primary ACL reconstruction and underwent surgery using a bone-patellar tendon-bone (BPTB) graft. The participants underwent three measurements: before ACL reconstruction, 3 months and 6 months after ACL reconstruction, respectively.

All procedures conducted in this study were anonymous and in accordance with the Declaration of Helsinki (25). The Ethical Committee of Masaryk University approved this study. All participants were informed about the aims and procedures of the study and gave written informed consent before the first measurement.

### 2.2 Plantar pressure analysis

To assess plantar pressure distribution, we used a Emed XL pressure platform (Novel GmbH, Germany; number of sensors: 25,344; sampling rate: 100 Hz; sensor area: 144 cm × 44 cm). The total walkway was 8.5 m in length (1.5 m platform and 4.5 m the Emed sidewalk placed before and after the platform). Every participant received instructions to walk at self-selected walking speed with gaze straight ahead on the platform, all while being barefoot. Following the signal, participants walked from the starting point to the finishing line spanning a distance of 8.5 m, after which they walk back outside the platform back on the starting point. This protocol was repeated until at least 5 valid footprints were recorded for each foot.

The Emed software itself divides the dynamic plantar pressure impressions of both feet separately into 11 different areas: Total Object (TO), hindfoot (HF), midfoot (MF), first metatarsal head (MH1), second metatarsal head (MH2), third metatarsal head (MH3), fourth metatarsal head (MH4), fifth metatarsal head (MH5), big toe (BT), second toe (ST), and toes 3–5 (T345). For each of these areas, maximum force (*N*), maximum force normalized to body weight (%BW), peak pressure (kPa), Contact time (*p*) (%ROP) (% of the rolling process) and contact area (cm^2^) were analyzed.

### 2.3 Muscle strength testing

To measure knee muscle strength, we used a calibrated isokinetic dynamometer (Humac Norm, Computer Sports Medicine, Inc., Stoughton, MA, USA). Participants were seated in the dynamometer chair with the backrest angled at 85°, and the dynamometer pad was positioned approximately 3 cm above the lateral malleolus. The knee joint axis was carefully aligned with the dynamometer's mechanical axis. Testing was initiated with the unaffected limb. To warm up and familiarize themselves with the procedure, participants completed five submaximal practice trials for each movement. After a 30 s rest, concentric isokinetic knee extension and flexion were assessed at an angular velocity of 60 degrees per second (60°/s^−1^). Each movement involved five maximal repetitions across a 90-degree range of motion, from full knee extension (0°) to 90° of knee flexion. Maximal knee extensor and flexor strength was determined by recording the peak torque (in Newton meters, Nm) during the isokinetic concentric contraction. Verbal encouragement was provided by the investigator to help participants reach their maximum strength, and they were not allowed to view the screen during the test. Gravity correction was performed before each test to ensure precise measurements. Peak torque values (Nm) obtained from the isokinetic strength testing were normalized to each participant's body mass (Nm/kg).

### 2.4 Anthropometric characteristics equipment

The participants' anthropometric characteristics were assessed using a digital scale (Seca 285, Hamburg, Germany) to measure standing height (in cm) and mass (in kg).

### 2.5 Data analysis

Due to the non-normal distribution of the data the Friedman test with Kendall's W was performed, and for statistically significant results, *post hoc* comparisons were conducted using Conover's method with Bonferroni correction. To examine the relationships between variables derived from plantar pressure analysis and the relative muscular strength a correlation analysis was conducted using Spearman's *ρ* and Fisher's Z. Spearman's correlation coefficients were interpreted according to the thresholds outlined: Very weak (*ρ* ≤ 0.199), Weak (*ρ* ≤ 0.399), Moderate (*ρ* ≤ 0.599), Strong (*ρ* ≤ 0.799), Very strong (*ρ* ≤ 1.0) (26).

Statistical analyses were carried out using IBM SPSS (version 29.0.0) and JASP (version 0.18.3). The significance level was set at 0.05, ensuring a rigorous framework for data analysis and supporting the reliability and validity of the findings.

## 3 Results

Although the final analysis was conducted using the non-parametric Friedman test, an *a priori* power analysis was performed using a repeated-measures ANOVA within-subjects design in G*Power 3.1.9.4, as this serves as an appropriate parametric approximation in the absence of a direct implementation for non-parametric tests. Based on an anticipated large effect size (*f* = 1.60), an alpha level of 0.05, a desired power of 0.95, three repeated measurements, and a moderate correlation among repeated measures (*r* = 0.50), the analysis indicated that a total sample size of 3 participants would be sufficient to detect the hypothesized effect. This corresponds to a noncentrality parameter *λ* = 46.29 and a critical *F*-value of 6.94, yielding an actual power of 0.977. These results confirm that the sample size used in the study (*n* = 10) provided adequate statistical power for the within-subject comparisons.

**Figure 1:**
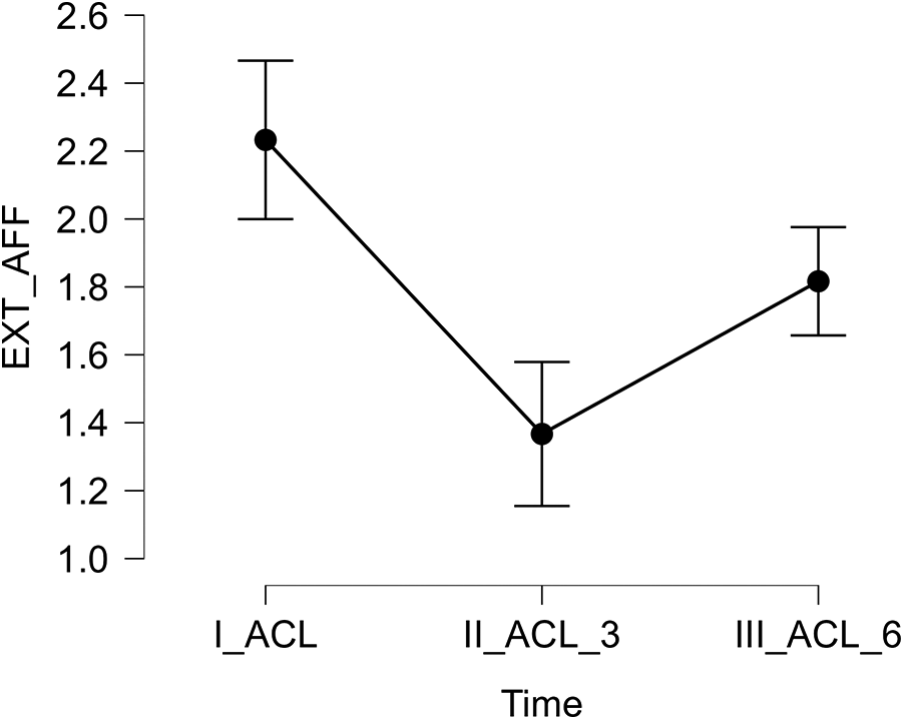
Relative strength of the extensor muscles of the affected limb measured preoperatively (I_ACL), three months postoperatively (II_ACL_3), and six months postoperatively (III_ACL_6).

**Figure 2:**
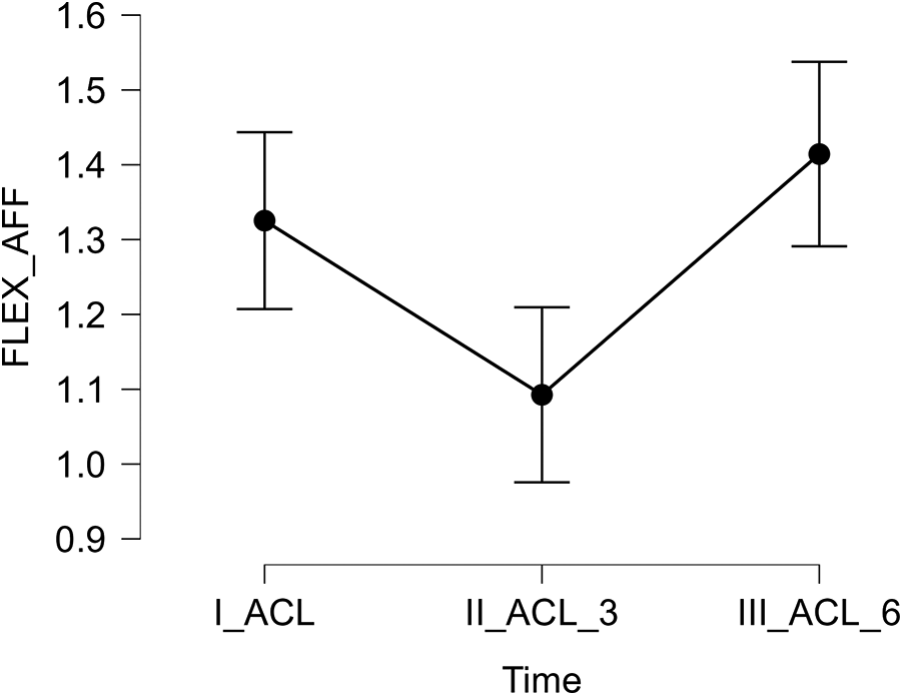
Relative strength of the flexor muscles of the affected limb measured preoperatively (I_ACL), three months postoperatively (II_ACL_3), and six months postoperatively (III_ACL_6).

Significant differences in isokinetic muscle strength of the knee extensors and flexors were identified between the affected and unaffected limbs. A marked reduction in extensor strength was observed at three and six months postoperatively compared to the preoperative baseline (both *p* < .001). Additionally, both extensor (*p* < .001) and flexor (*p* = .005) strength significantly improved at six months relative to the three-month follow-up. These results are presented in Table 1 and Figures 1, 2.

**Table 1:** Repeated measures comparison of muscular strength.

Variable	*n*	Muscular strength [MED (Q_1_–Q_3_)]			*Χ* ^2^	*df*	*p*	*W*	Post Hoc
		I_ACL	II_ACL_3	III_ACL_6					
EXT_AFF	10	2.46 (1.67–2.82)	1.42 (1.04–1.54)	1.94 (1.23–2.41)	18.20	2	<.001	0.91	I > II; I > III; II < III
EXT_UAFF	10	2.80 (2.37–2.99)	2.78 (2.19–3.06)	2.63 (2.15–3.24)	0.80	2	0.670	0.04	
FLEX_AFF	10	1.55 (0.90–1.61)	1.22 (0.73–1.32)	1.55 (1.25–1.77)	8.60	2	0.014	0.43	II < III
FLEX_UAFF	10	1.54 (1.24–1.82)	1.45 (1.31–1.84)	1.64 (1.26–1.88)	5.60	2	0.061	0.28	

I_ACL, the preoperative measurement; II_ACL_3, the three months postoperative measurement; III_ACL_6, the six months postoperative measurement; EXT_AFF, relative strength of extensor muscles of the affected extremity; EXT_UAFF, relative strength of extensor muscles of the unaffected extremity; FLEX_AFF, relative strength of flexor muscles of the affected extremity; FLEX_UAFF, relative strength of flexor muscles of the unaffected extremity.

Table 2 presents the plantar pressure analysis across 11 predefined foot regions and five variables. Statistically significant findings were observed in the following parameters: hindfoot contact area [*χ*^2^(2) = 6.20, *p* = .045, *w* = 0.31], midfoot maximum force normalized to body weight (BW) [*χ*^2^(2) = 7.40, *p* = .025, *w* = 0.37], and peak pressure of the fifth metatarsal head [*χ*^2^(2) = 6.20, *p* = .045, *w* = 0.31].

**Table 2:** Plantar pressure distribution of foot across the eleven different areas.

Area	Measurement	*n*	*Χ* ^2^	*df*	*p*	*W*
Total object (TO)	Maximum force (*N*)	10	4.15	2	.125	0.21
	Maximum force (normalized to BW) (%BW)	10	0.20	2	.905	0.01
	Peak pressure (kPa)	10	2.40	2	.301	0.12
	Contact time (*p*) (%ROP)	10	0.01	2	.999	0.01
	Contact area (cm^2^)	10	2.60	2	.273	0.13
Hindfoot (HF)	Maximum force (*N*)	10	0.20	2	.905	0.01
	Maximum force (normalized to BW) (%BW)	10	0.80	2	.670	0.40
	Peak pressure (kPa)	10	0.20	2	.905	0.01
	Contact time (*p*) (%ROP)	10	0.60	2	.741	0.03
	Contact area (cm^2^)	10	6.20	2	.045	0.31
Midfoot (MF)	Maximum force (*N*)	10	3.80	2	.150	0.19
	Maximum force (normalized to BW) (%BW)	10	7.40	2	.025	0.37
	Peak pressure (kPa)	10	3.44	2	.179	0.17
	Contact time (p) (%ROP)	10	1.40	2	.497	0.07
	Contact area (cm^2^)	10	1.28	2	.527	0.06
First metatarsal head (MH1)	Maximum force (*N*)	10	2.40	2	.301	0.12
	Maximum force (normalized to BW) (%BW)	10	2.60	2	.273	0.13
	Peak pressure (kPa)	10	1.40	2	.497	0.07
	Contact time (*p*) (%ROP)	10	0.20	2	.905	0.01
	Contact area (cm^2^)	10	2.400	2	.301	0.12
Second metatarsal head (MH2)	Maximum force (*N*)	10	2.60	2	.273	1.13
	Maximum force (normalized to BW) (%BW)	10	0.20	2	.905	0.01
	Peak pressure (kPa)	10	0.20	2	.905	0.01
	Contact time (*p*) (%ROP)	10	0.20	2	.905	0.01
	Contact area (cm^2^)	10	1.59	2	.452	0.08
Third metatarsal head (MH3)	Maximum force (*N*)	10	2.21	2	.332	0.11
	Maximum force (normalized to BW) (%BW)	10	1.39	2	.500	0.07
	Peak pressure (kPa)	10	0.20	2	.905	0.01
	Contact time (*p*) (%ROP)	10	0.80	2	.670	0.04
	Contact area (cm^2^)	10	1.39	2	.500	0.07
Forth metatarsal head (MH4)	Maximum force (*N*)	10	3.20	2	.202	0.16
	Maximum force (normalized to BW) (%BW)	10	1.08	2	.584	0.05
	Peak pressure (kPa)	10	0.80	2	.670	0.04
	Contact time (*p*) (%ROP)	10	0.60	2	.741	0.03
	Contact area (cm^2^)	10	0.68	2	.710	0.03
Fifth metatarsal head (MH5)	Maximum force (*N*)	10	2.60	2	.273	0.13
	Maximum force (normalized to BW) (%BW)	10	2.60	2	.273	0.13
	Peak pressure (kPa)	10	6.20	2	.045	0.31
	Contact time (*p*) (%ROP)	10	1.11	2	.575	0.06
	Contact area (cm^2^)	10	0.36	2	.836	0.02
Big toe (BT)	Maximum force (*N*)	10	2.92	2	.232	0.15
	Maximum force (normalized to BW) (%BW)	10	2.92	2	.232	0.15
	Peak pressure (kPa)	10	0.80	2	.670	0.04
	Contact time (*p*) (%ROP)	10	5.60	2	.061	0.28
	Contact area (cm^2^)	10	2.92	2	.232	0.15
Second toe (ST)	Maximum force (*N*)	10	1.40	2	.497	0.07
	Maximum force (normalized to BW) (%BW)	10	1.90	2	.388	0.10
	Peak pressure (kPa)	10	1.80	2	.407	0.09
	Contact time (*p*) (%ROP)	10	1.40	2	.497	0.07
	Contact area (cm^2^)	10	0.60	2	.741	0.03
Toes 345 (T345)	Maximum force (*N*)	10	2.60	2	.273	0.13
	Maximum force (normalized to BW) (%BW)	10	3.43	2	.179	0.17
	Peak pressure (kPa)	10	2.60	2	.273	0.13
	Contact time (*p*) (%ROP)	10	0.60	2	.741	0.03
	Contact area (cm^2^)	10	1.40	2	.497	0.07

Post hoc analysis using Conover's test with Bonferroni correction revealed significant differences across all three plantar pressure parameters. In the hindfoot region, the preoperative contact area was significantly greater than at six months postoperatively (*p* = .035). For the midfoot, maximum force normalized to body weight was significantly lower at both three months (*p* = .047) and six months (*p* = .026) compared to the preoperative values. Similarly, peak pressure under the fifth metatarsal head was significantly reduced at six months postoperatively relative to baseline (*p* = .035).

Table 3 presents the results of the correlation analysis between muscle strength (knee extensors and flexors) and plantar pressure parameters, including hindfoot contact area, midfoot maximum force, and peak pressure under the fifth metatarsal head. No statistically significant relationships were observed between muscle strength and any of the plantar pressure variables.

**Table 3:** Correlation between isokinetic strength variables and plantar pressure parameters.

Variables	*ρ*	95% CI	*p*
EXT_AFF vs. Hindfoot Contact Area	0.159	−0.178–0.492	0.402
EXT_AFF vs. Midfoot Max Force (% BW)	−0.223	−0.521–0.130	0.236
EXT_AFF vs. MH5 Peak Pressure	0.23	−0.185–0.650	0.221
FLEX_AFF vs. Hindfoot Contact Area	0.116	−0.281–0.494	0.542
FLEX_AFF vs. Midfoot Max Force (% BW)	−0.097	−0.394–0.263	0.612
FLEX_AFF vs. MH5 Peak Pressure	0.106	−0.341–0.532	0.578
Hindfoot Contact Area vs. Midfoot Force	0.02	−0.365–0.382	0.918
Hindfoot Contact Area vs. MH5 Pressure	−0.15	−0.515–0.273	0.429
Midfoot Force vs. MH5 Peak Pressure	0.345	−0.040–0.634	0.062

MH5, fifth metatarsal head; % BW, percentage of body weight.

## 4 Discussion

This study aimed to examine the recovery of isokinetic knee muscle strength and dynamic plantar pressure distribution during barefoot level walking in individuals with unilateral ACL rupture, assessed at three time points: preoperatively, and at three and six months following ACL reconstruction using a BPTB graft. Special attention was paid to the evaluation of plantar loading patterns in anatomically defined foot regions and their relationship to knee extensor and flexor strength in the affected limb. The purpose of this study was to determine whether insufficient lower-limb muscle strength is associated with alterations in dynamic plantar pressure and asymmetrical gait patterns during early rehabilitation after ACL reconstruction.

We hypothesized that limited quadriceps and hamstring strength following ACL reconstruction would be associated with persistent abnormalities in plantar pressure distribution, particularly in the hindfoot and midfoot regions, reflecting compensatory gait mechanisms and incomplete neuromuscular recovery. Furthermore, we expected a significant correlation between lower-limb muscle strength and dynamic plantar pressure parameters, suggesting a functional relationship between isokinetic strength and gait-related loading patterns.

The results of our study partially supported this hypothesis. At three and six months post-ACL reconstruction, our findings revealed persistent quadriceps strength deficits compared to preoperative levels. Notably, a significant increase in extensor strength was observed between the three- and six-month follow-ups, suggesting gradual muscular recovery. A similar, although slightly less pronounced, trend was observed in hamstring strength, with a significant improvement from three to six months postoperatively. These results align with previous studies that have consistently reported substantial quadriceps weakness in the early postoperative phase, particularly among individuals who underwent reconstruction using a BPTB graft (27, 28). Given the fundamental role of quadriceps strength in restoring knee joint stability, promoting gait normalization, and reducing reinjury risk, these findings emphasize the importance of progressive strength rehabilitation. Despite the observed improvements, the presence of strength deficits at six months suggests that many patients may not achieve sufficient neuromuscular readiness for safe return to sport within this time frame.

While we observed persistent deviations in plantar pressure distribution—particularly a significantly reduced contact area in the hindfoot at six months postoperatively—our results further demonstrated significantly lower midfoot maximum force at three and six months postoperatively compared to preoperative values. Additionally, peak pressure under the fifth metatarsal head remained significantly reduced at the six months postoperatively. These findings indicate persistent alterations in plantar loading that are not limited to the early rehabilitation phase but may reflect prolonged disruptions in normal gait mechanics.

Our results are further supported by the recent study by Liu et al. (29), who evaluated lower limb loading symmetry after ACL reconstruction using a pressure-sensing walkway. They found that weight-bearing asymmetries between the operated and non-operated limbs persisted at six months postoperatively, particularly during gait. Although their methodology differs from ours, their findings align with our observation of reduced hindfoot loading and continued asymmetry in plantar pressure distribution at six months. These consistent findings underscore the potential value of pressure-based gait analysis in complementing conventional clinical assessments, particularly in the context of return-to-sport decision-making.

Our findings are partially consistent with those of Çetın et al. (22), who reported altered plantar pressure distributions in ACL-deficient patients, with decreased hindfoot and increased midfoot loading on the injured limb. Following ACL reconstruction, their patients demonstrated a significant postoperative increase in hindfoot loading, approaching values observed in healthy controls at a mean follow-up of 14.5 months. In contrast, our study found that hindfoot pressure remained significantly reduced even six months postoperatively, indicating a prolonged asymmetry in rearfoot loading. These discrepancies may be attributed to differences in follow-up duration, rehabilitation protocols, or assessment methods. Nonetheless, both studies highlight the relevance of plantar pressure analysis in monitoring functional recovery and suggest that normalization of gait-related plantar loading patterns may extend beyond the early postoperative period. Furthermore, the increase in hindfoot pressure indicates more efficient center of gravity transfer, facilitating smoother and more coordinated gait patterns that support improved knee control and dynamic balance (30, 31).

Our results can also be compared with the findings of (32), who reported altered plantar pressure distribution in ACL-deficient patients characterized by reduced heel loading and earlier forefoot contact during gait. These patterns were interpreted as compensatory mechanisms aimed at minimizing anterior tibial translation and protecting the injured knee joint. In line with their observations, our study also identified persistent reductions in hindfoot loading even six months postoperatively, suggesting that altered gait mechanics may remain long after surgical reconstruction. While their study focused on the preoperative phase, our findings further contribute to this understanding by demonstrating that such compensatory strategies may persist beyond reconstruction, despite restored structural stability. This highlights the importance of targeted rehabilitation strategies not only for strength recovery but also for the re-establishment of physiological gait patterns.

Our findings contrast with those of Mittlmeier et al. (33), who demonstrated that plantar pressure distribution in the hindfoot area normalized as early as 12 weeks postoperatively, with no significant differences observed between the operated and non-operated limbs during level walking. In our study, however, significant asymmetry in hindfoot loading persisted even at six months postoperatively, as evidenced by a reduced contact area compared to preoperative values. This suggests that full restoration of symmetrical heel loading may take longer than previously reported, or may vary depending on individual recovery trajectories or rehabilitation protocols.

Furthermore, the correlation analysis revealed no statistically significant relationships between isokinetic strength of knee extensors or flexors and plantar pressure parameters, including hindfoot contact area, midfoot maximum force, and peak pressure under the fifth metatarsal head. This absence of correlation suggests that dynamic plantar loading characteristics may not be directly modulated by isolated measures of muscular strength. Instead, it is plausible that other factors such as neuromuscular coordination, proprioceptive function, or central motor control strategies play a more prominent role in mediating gait adaptations post-reconstruction. These findings contrast with those of Mittlmeier et al. (33), who reported a significant negative correlation (Spearman's *ρ* = –0.68, *p* < 0.05) between quadriceps strength deficits and impulse transfer through the injured limb in ACL-deficient patients. According to their results, weaker quadriceps strength was associated with reduced plantar loading, reflecting a compensatory unloading strategy. The discrepancy between our findings and those of Mittlmeier et al. (33) may be explained, at least in part, by methodological differences. While their analysis relied on relative impulse-based indices—such as total impulse, rearfoot impulse (M1 + M2), and various forefoot impulse ratios—our study focused on absolute values of plantar pressure in anatomically defined regions. Additionally, the measurement technology used in our study offered higher spatial resolution and sampling frequency, in contrast to earlier systems with lower sensor density. This enhanced sensitivity may have captured subtle variations in plantar loading undetectable by earlier systems. Our data therefore suggest that, at six months postoperatively, plantar pressure distribution may not be directly determined by isolated muscle strength and that gait symmetry restoration likely involves more complex neuromuscular recovery mechanisms.

These findings suggest that plantar pressure distribution analysis may serve as a valuable supplementary criterion for RTS decision-making, particularly by emphasizing the normalization of hindfoot pressure symmetry between the involved and uninvolved limbs. The persistent deviations observed in rearfoot loading patterns at six months postoperatively indicate that gait adaptations may extend beyond the typical RTS timeframe, even in the absence of significant residual strength deficits. However, our results also demonstrate that dynamic plantar pressure variables alone are insufficient to accurately estimate knee extensor or flexor muscle recovery. This lack of correlation implies that other neuromuscular or proprioceptive mechanisms likely contribute to gait compensation strategies and should be considered in comprehensive rehabilitation evaluation.

This study has several limitations. First, the relatively small sample size (*n* = 10) may reduce the statistical power and generalizability of our findings. Second, while repeated measures were taken, the six-month follow-up may not have been long enough to capture full functional recovery or the resolution of gait asymmetries. Future studies should aim to include longer follow-up periods (e.g., ≥12 months), larger and more diverse cohorts, and combine plantar pressure analysis with electromyographic or kinematic data to better elucidate the complex neuromuscular adaptations following ACL reconstruction.

In conclusion, the integration of dynamic plantar pressure analysis into RTS protocols may offer clinicians a more nuanced understanding of gait restoration, beyond standard strength or hop tests. Importantly, it could help detect subclinical asymmetries that persist despite the apparent restoration of muscle strength, thereby identifying patients at elevated risk of reinjury. Future interventions should explore whether individualized gait retraining and targeted strategies aimed at both proximal (quadriceps) and distal (foot-loading) function can accelerate recovery and improve long-term outcomes after ACL reconstruction.

## Data Availability

The raw data supporting the conclusions of this article will be made available by the authors, without undue reservation.
